# The Prevention of Infectious Disease by Inoculation

**Published:** 1881-01

**Authors:** 


### EDITORIAL DEPARTMENT.

### THE PREVENTION OF INFECTIOUS DISEASES BY INOCULATION.

THE question whether acute infectious diseases can be prevented or mitigated in violence by inoculation has lately been brought into great prominence. Holland now announces that by inoculation and isolation it has rid itself of pleuro-pneumonia. If this is actually the case—and the facts are vouched for by Fleming—the question of inoculation for pleuro-pneumonia deserves further study from American veterinarians, by whom the measure is now generally condemned. But, in addition to the above reports, there have been a number of experimental investigations, which seem to throw some light upon the subject of inoculation, as a preventive measure, in many other diseases. Toussaint has experimented with the virus of anthrax, and claims to have modified it so that its inoculation causes a mild protective disease in sheep. Burdon-Sanderson and Greenfield have conducted similar investigations upon heifers, and claim similar results. Finally, Pasteur has announced that he has discovered a method by which he can obtain a true "vaccine matter" for fowl-cholera; that is to say, a material which is to the cholera what vaccinia is to small-pox.

We venture to give some details of M. Pasteur's alleged discovery, because, though we have but little faith in his bacteria, we do not consider it impossible that a "vaccine" may have been discovered.

M. Toussaint some time ago announced that he had discovered the specific germ of fowl-cholera in the shape of a microscopic organism, oval-shaped, multiplying transversely and capable of cultivation in certain fluids. Pasteur confirmed this discovery, and added to it by numerous experiments of his own. These resulted in the obtaining, by cultivation, a modified or weakened form of the virus, which when inoculated into healthy fowls protects them, as he claims, against the cholera. The way in which he obtained this protective matter was as follows : There are, he says, two kinds of chicken-cholera, an acute and a chronic. In the latter form, the poison seems to be stored up in the organs of the animal for some time; it finally works into the general system, and it then acts upon it very virulently, rapidly causing death. The acute disease is of a milder type, and does not always kill. Pasteur takes some of the secretions of the fowl containing the specific germs. These he cultivates in a decoction of the fowl's muscle. The organisms rapidly multiply for a certain time, and the mixture becomes turbid. It then clears up gradually, the bacteria settling to the bottom. It is found that the fluid will now allow of the development of no more of the specific organisms, even if a fresh supply of the most virulent kind is put in. The settled fluid is then allowed to stand for eight or ten months; at the end of that time, some of it is taken and placed in a fresh muscle decoction, when development and turbidity again appear. Now, if some of this second culture is inoculated in a fowl, it causes a very mild form of the disease, which, however, protects the animal against any further attacks, even when the strongest virus is employed against it. This attenuation and weakening is produced by the long interval between the cultures, as is shown from the fact that, if cultures succeed each other immediately, their virulence is unimpaired. Furthermore, the special effect is thought by Pasteur to be due to the action of the oxygen upon the virus; for if the fluids are kept during the intervals between the cultures in hermetically sealed tubes, they are as strong at the end as at the beginning of the experiments, no matter how long the time. These experiments of Pasteur are certainly very striking, and we hope they may prove to be true. But even if incorrect in many of his theoretical deductions, the work of Pasteur and other investigators points strongly to the possibility of securing a protective virus for many infectious diseases. It is a question that certainly deserves the fullest attention from pathologists and veterinarians.

